# IS*903B*-mediated *cirA* inactivation confers cefiderocol resistance and adaptive fitness in carbapenem-resistant *Klebsiella pneumoniae*

**DOI:** 10.1080/22221751.2026.2736341

**Published:** 2026-09-25

**Authors:** Yuxuan Liu, Xinyue Dai, Hanxu Hong, Chuwen Zhao, Yun Cao, Linping Fan, Yang Liu

**Affiliations:** a Department of Clinical Laboratory, The First Affiliated Hospital of Nanchang University, Nanchang, People’s Republic of China; b School of Public Health, Jiangxi Medical College, Nanchang University, Nanchang, People’s Republic of China; c First Clinical Medical College, Nanchang University, Nanchang, People’s Republic of China; d Center for Molecular Diagnosis and Precision Medicine, The First Affiliated Hospital, Jiangxi Medical College, Nanchang University, Nanchang, People’s Republic of China; e Jiangxi Medical Center for Critical Public Health Events, The First Affiliated Hospital, Jiangxi Medical College, Nanchang University, Nanchang, People’s Republic of China

**Keywords:** *Klebsiella pneumoniae*, cefiderocol resistance, *cirA*, IS9*03B*, iron-limited adaptation

## Abstract

Cefiderocol (FDC) is a last-resort antimicrobial for the treatment of carbapenem-resistant *Klebsiella pneumoniae* (CRKP). Although *cirA* inactivation is a recognized mechanism of FDC resistance, whether different forms of *cirA* disruption influence bacterial adaptation and persistence remains unclear. By analyzing 1,965 publicly available long-read genomes and 480 clinical isolates, we found that *cirA* loss-of-function mutations were rare (1.1%) but accumulated over time among isolates from China, with four independently emerged *cirA*-inactivated ST11 isolates identified across distinct phylogenetic branches. Functional characterization of a representative ST11-KL64 clinical isolate showed that IS*903B*-mediated *cirA* inactivation conferred high-level FDC resistance (minimum inhibitory concentration, 128 μg/mL), which was reversed by *cirA* complementation. Functional assays further demonstrated that, unlike complete *cirA* deletion, IS*903B*-mediated *cirA* inactivation enhanced bacterial adaptation under iron-limited conditions, including competitive fitness, plasmid stability, and host-associated survival. Transcriptomic, enzymatic, and genetic analyses supported distinct Fe-S-associated metabolic remodelling in *cirA*::IS*903B*, while deletion of *sufS* attenuated these adaptive phenotypes. Construction of *cirA*::IS*903B* derivatives in multiple independent ST11 clinical isolates reproduced these adaptive phenotypes across diverse genetic backgrounds. Together, these findings demonstrate that IS*903B*-mediated *cirA* inactivation not only confers high-level FDC resistance but also promotes bacterial adaptation through mechanisms distinct from complete *cirA* deletion. The combination of resistance, enhanced adaptation, and increased plasmid stability suggests that specific resistance-associated *cirA* mutations may facilitate the persistence and dissemination of high-risk CRKP clones. Monitoring *cirA* integrity and IS insertion status may help identify FDC-resistant lineages with increased dissemination potential.

## GRAPHICAL ABSTRACT

**Figure:**
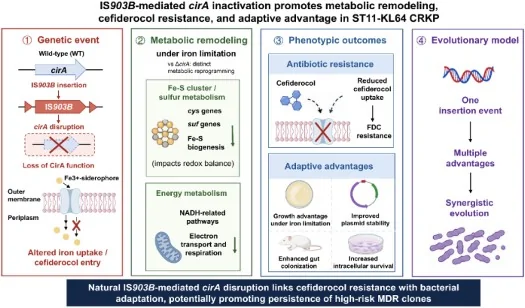

## Introduction

*Klebsiella pneumoniae* is a major pathogen responsible for hospital-acquired infections and can cause a wide range of infectious diseases [1]. In recent years, carbapenem-resistant *K. pneumoniae* (CRKP) has spread rapidly worldwide, posing a serious public health threat and further limiting available antimicrobial therapies [2–4]. Cefiderocol (FDC) is a novel siderophore cephalosporin that enters bacterial cells through a “Trojan horse” strategy by hijacking iron uptake systems. It exhibits potent activity against multidrug-resistant Gram-negative bacteria and is considered an important last-resort agent for treating complicated resistant infections [5,6]. Nevertheless, the emergence of FDC-nonsusceptible CRKP isolates has raised growing clinical concerns, despite the generally high activity of FDC against carbapenem-resistant pathogens [7–9].

Current studies suggest that FDC resistance is mediated by multiple mechanisms, including increased β-lactamase expression, altered outer membrane permeability, and changes in the structure or expression of iron uptake receptors [10–13]. Among these mechanisms, *cirA*, which encodes a catecholate siderophore receptor, serves as a key entry pathway for FDC, and its mutation or inactivation can markedly reduce FDC uptake efficiency [14,15]. Previous studies have shown that truncation, frameshift, or premature stop mutations in *cirA* can result in loss of function and reduced FDC susceptibility [16,17]. However, the distribution, evolutionary patterns, and potential biological significance of *cirA* inactivation in *K. pneumoniae* populations remain poorly understood. Resistance mutations have traditionally been considered to impose fitness costs [18]. However, recent studies suggest that bacteria may alleviate these costs through metabolic reprogramming or regulatory network remodelling and may even acquire additional adaptive advantages [19–22]. As mobile transposable elements, insertion sequences (ISs) can disrupt gene structures and alter host adaptation by affecting neighbouring gene expression and local regulatory networks [23–25].

Sequence type 11 capsular locus 64 (ST11-KL64) is one of the predominant high-risk CRKP clones in China and commonly carries resistance genes such as *bla*_KPC_ and *bla*_NDM_. Acquisition of virulence plasmids may further drive its evolution into hypervirulent CRKP [26]. In this clone, our previous genomic surveillance identified multiple independently emerging *cirA* loss-of-function mutations distributed across distinct evolutionary branches, suggesting possible positive selection for this trait. Although *cirA* inactivation has been repeatedly associated with cefiderocol resistance, whether different forms of *cirA* disruption exert distinct biological consequences beyond resistance remains largely unknown. In this study, an ST11-KL64 clinical isolate carrying IS*903B*-mediated *cirA* inactivation was used to compare the resistance and fitness phenotypes associated with different forms of *cirA* loss of function through isogenic strain construction. Transcriptomic analysis was further performed to preliminarily investigate potentially involved alterations in metabolic pathways.

## Materials and methods

### Global collection and local surveillance of *K. pneumoniae*

Publicly available long-read *K. pneumoniae* genomes deposited in the NCBI database up to February 2024 were collected for genomic analysis, yielding 1,965 non-duplicate genomes. In parallel, 480 clinical *K. pneumoniae* isolates were collected from a tertiary hospital in Jiangxi, China. *cirA* variants were identified by PCR and Sanger sequencing. Primer sequences are listed in Supplementary Table 1.

### Antimicrobial susceptibility testing (AST)

The minimum inhibitory concentration (MIC) of FDC was determined by broth microdilution in iron-depleted cation-adjusted Mueller–Hinton broth (ID-CAMHB) as previously described 23 and interpreted according to CLSI guidelines [27].

### Construction of *cirA* deletion and complemented strains

A CRISPR/Cas9 system was used to generate the complete *cirA* deletion mutant (Δ*cirA*) in the K2084 (*cirA*::IS*903B*) background. For complementation, the intact *cirA* gene was cloned into an expression vector and introduced into the corresponding strain. Detailed construction procedures and primer sequences are provided in the Supplementary Materials and Supplementary Table 1.

### Whole-genome sequencing

Whole-genome sequencing, genome assembly, phylogenetic analysis, and mobile genetic element prediction were performed as described in the Supplementary Materials.

### Transcriptomic analysis and RT-qPCR validation

Transcriptomic sequencing and RT-qPCR validation were performed under LB and ID-CAMHB conditions as described in the Supplementary Materials. Relative gene expression levels were normalized to 16S rRNA and calculated using the 2^^−ΔΔCT^ method. Primer sequences are listed in Supplementary Table 1.

### Growth curve and time-kill assays

Growth curves were determined in LB or ID-CAMHB by monitoring OD540 over time. Time-kill assays were performed in ID-CAMHB with different concentrations of FDC, with or without avibactam (4 μg/mL). Bacterial survival was quantified by CFU enumeration, and synergy and bactericidal activity were defined as reductions of ≥2 log₁₀ CFU/mL and ≥3 log₁₀ CFU/mL, respectively.

### *In vitro* competition and plasmid stability assays

Pairwise competition assays were performed in LB and ID-CAMHB, and relative fitness (w) was calculated based on changes in strain abundance over time. For plasmid stability assays, strains were serially passaged in LB, ID-CAMHB, or LB supplemented with meropenem (2 μg/mL), and plasmid retention rates were determined by selective plating.

### Intestinal colonization model

Mouse intestinal colonization assays were performed as described in the Supplementary Materials. All animal experiments were approved by the Ethics Committee of the First Affiliated Hospital of Nanchang University.

### Macrophage phagocytosis assay

Macrophage intracellular survival assays using RAW 264.7 cells were performed as described in the Supplementary Materials.

### Oxidative stress-associated phenotypes and aconitase activity assays

2,2′-azinobis-(3-ethylbenzothiazoline-6-sulfonic acid) (ABTS) radical scavenging activity, superoxide dismutase (SOD) activity, intracellular reactive oxygen species (ROS) levels, and aconitase activity were determined as described in the Supplementary Materials.

### Statistical analysis

Statistical analyses were performed using unpaired Student’s t test or one-way analysis of variance (ANOVA). A *P* value < 0.05 was considered statistically significant.

## Results

### Global variation landscape and evolutionary trends of *cirA* in *K. pneumoniae*

To systematically characterize the global variation landscape of *cirA* in *K. pneumoniae*, we screened 76,828 genome assemblies deposited in the NCBI database up to February 2024 and obtained 1,965 non-duplicate genomes generated by long-read sequencing, covering isolates from 60 countries worldwide and 29 provinces in China. Most isolates originated from China (870/1,965, 44.3%), followed by the United States (225/1,965, 11.5%), Australia (120/1,965, 6.1%), and the United Kingdom (81/1,965, 4.1%). Within China, isolates were mainly distributed in Zhejiang Province (108/870, 12.4%), Taiwan Province (72/870, 8.3%), Shanghai (63/870, 7.2%), and Sichuan Province (62/870, 7.1%) (Figure 1A). Using the *cirA* sequence of strain ATCC 13883 (accession no. CP040993.1) as the reference, we comprehensively analyzed the structural variation landscape, including amino acid substitutions, gene truncations, and insertion events. High-frequency amino acid substitutions were mainly observed at D558N (522/1,965, 26.6%), Y183W (198/1,965, 10.1%), T172A (94/1,965, 4.8%), V237I (82/1,965, 4.2%), and E81V (48/1,965, 2.4%). In contrast, truncation and insertion events leading to *cirA* inactivation were rare, with only 21 isolates identified (Supplementary Table 2), accounting for 1.1% of all isolates (Figure 1C).

**Figure 1.**
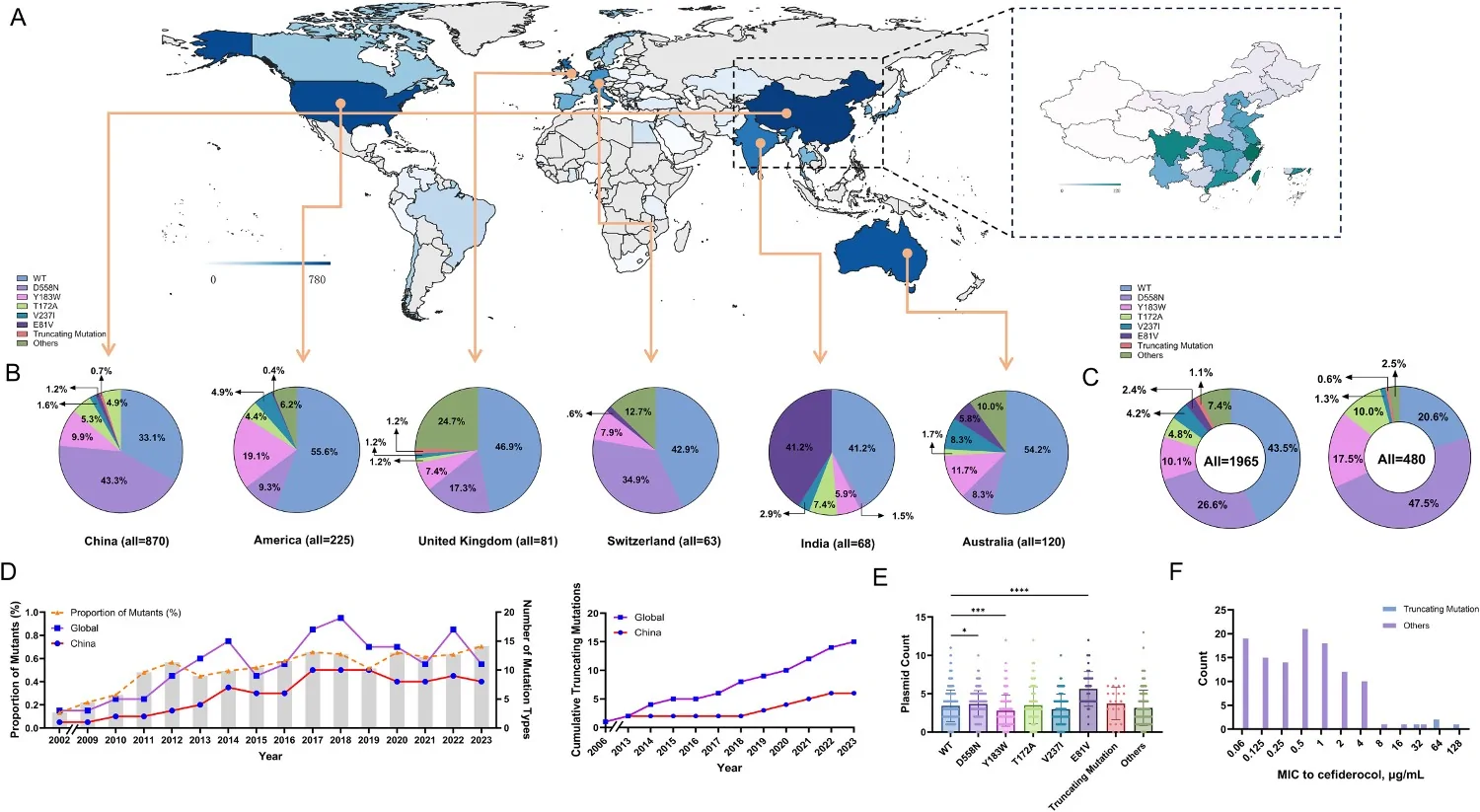
Global and local distribution and mutation landscape of the *cirA* gene in *Klebsiella pneumoniae*. (A) Geographic distribution of 1,965 third-generation sequenced *K. pneumoniae* isolates worldwide up to February 2024. (B) Pie charts showing the composition of *cirA* amino acid variants among the six countries with the largest sample sizes. (C) Overall distribution of *cirA* amino acid variants in global and local isolates. (D) Temporal changes in the overall proportions and diversity of *cirA* mutation types across different years globally and in China (left), and cumulative frequency of truncation mutations over time (right). (E) Plasmid burdens among strains carrying different *cirA* variants. *, *P* < 0.05; ***, *P* < 0.001; ****, *P* < 0.0001. (F) Comparison of FDC resistance levels between clinical isolates carrying truncated *cirA* and randomly selected non-truncated isolates.

Analysis of isolates with available collection dates showed that the proportion of isolates carrying *cirA* amino acid substitutions increased continuously after 2010, accompanied by progressively greater mutational diversity. Globally, truncation mutations became increasingly detectable after 2014, whereas in China, these mutations were mainly identified after 2018 (Figure 1D). In addition, the *cirA* variation landscape differed substantially across geographic regions. In Chinese isolates, the D558N substitution was predominant and occurred more frequently than the wild-type genotype (43.4% vs. 33.1%), whereas the wild-type genotype remained dominant in other major countries (Figure 1B). Notably, truncation mutations were identified only in isolates from China and the United Kingdom and showed a temporal accumulation trend.

Among 480 *K. pneumoniae* clinical isolates collected from multiple centres in Jiangxi, China, the *cirA* variation landscape was generally consistent with that of the global dataset. Amino acid substitutions remained the predominant form of variation, whereas loss-of-function mutations were rare, accounting for only 0.63% (3/480) of isolates (Figure 1C). Further analysis showed that certain *cirA* amino acid variants, including D558N and Y183W, were associated with significantly different plasmid burdens compared with the wild-type genotype in the global collection (*P* < 0.05) (Figure 1E), suggesting variant-dependent differences in plasmid carriage. In addition, clinical isolates carrying truncated *cirA* exhibited higher levels of FDC resistance than those of 120 randomly selected non-truncated isolates (Figure 1F).

To further investigate the distribution of *cirA* loss-of-function mutations in high-risk clones, a core genome phylogeny was constructed for ST11 strains (Supplementary Figure 1). Among 478 ST11 isolates, only four carried *cirA* loss-of-function-associated variants, including one gene interruption, two premature stop mutations, and one small-fragment deletion. All four isolates were recovered from clinical samples in China between 2012 and 2022. These strains were distributed across distinct phylogenetic branches rather than clustering within a single lineage, suggesting that *cirA* inactivation likely emerged independently multiple times within the ST11 background and was repeatedly maintained through host- and environment-associated selective forces rather than driven by clonal expansion. Further analysis showed that these isolates were associated with distinct resistance backgrounds, with some carrying carbapenemase genes such as *bla*_KPC_ and *bla*_NDM_, suggesting that *cirA* inactivation may provide additional adaptive advantages in certain high-risk resistant lineages.

### Characteristics of IS*903B*-mediated *cirA* inactivation in clinical ST11 CRKP isolates

Although phylogenetic analysis suggested that *cirA* inactivation represents a rare but convergently evolved event that emerged independently multiple times within high-risk ST11 clones, whether this event is associated with fitness-related phenotypic changes required further functional validation. During clinical surveillance in Jiangxi, China, multiple forms of *cirA* loss-of-function mutations were identified, including premature stop mutations and IS insertions. To further investigate their functional effects, the representative high-risk strain K2084 (ST11-KL64), carrying IS*903B*-mediated *cirA* inactivation together with *bla*_KPC_, *bla*_NDM_, and a virulence plasmid, was selected for in-depth functional analysis. A complete *cirA* deletion mutant (Δ*cirA*) was further constructed in the same genetic background as a loss-of-function control to compare the biological effects of different forms of *cirA* inactivation.

K2084 was isolated from peritoneal secretion specimens collected from a patient at a tertiary hospital in Jiangxi, China. The patient had underlying medical conditions, including diabetes mellitus, and received multiple antimicrobial therapies during hospitalization, including ampicillin/sulbactam, meropenem, tigecycline, and polymyxin, but ultimately died. Although the patient had not received FDC or ceftazidime/avibactam during hospitalization, antimicrobial susceptibility testing showed that K2084 exhibited high-level FDC resistance (MIC = 128 μg/mL) and a typical multidrug-resistant (MDR) phenotype. Genomic analysis showed that, apart from *cirA*, other major siderophore receptor genes, including *fepA*, *fhuA*, *fhuE*, and *fiu*, as well as efflux pump-associated genes, remained intact without detectable mutations. Although K2084 carried multiple virulence-associated genes and exhibited a high virulence score, no hypervirulent phenotype was observed (Supplementary Figure 2).

The *cirA* gene was interrupted by the insertion sequence IS*903B* and was accompanied by a 9-bp target site duplication (TSD; CCGATGTGA), consistent with a typical transposition event. Functional annotation of the approximately 20 kb genomic region surrounding *cirA* revealed enrichment of multiple insertion sequences and transposase-associated genes, indicating a mobile genetic element-rich region with high transposition activity. In K2084, in addition to the IS*903B* insertion within *cirA*, additional insertion sequences were identified upstream of the gene, further supporting elevated transposition activity in this region. Besides *cirA*, this locus also contained genes associated with the phosphotransferase system-mediated fructose transport system, the Mgl galactose/glucose ATP-binding cassette transport system, and core metabolic functions including DNA repair and folate biosynthesis (Figure 2A).

**Figure 2.**
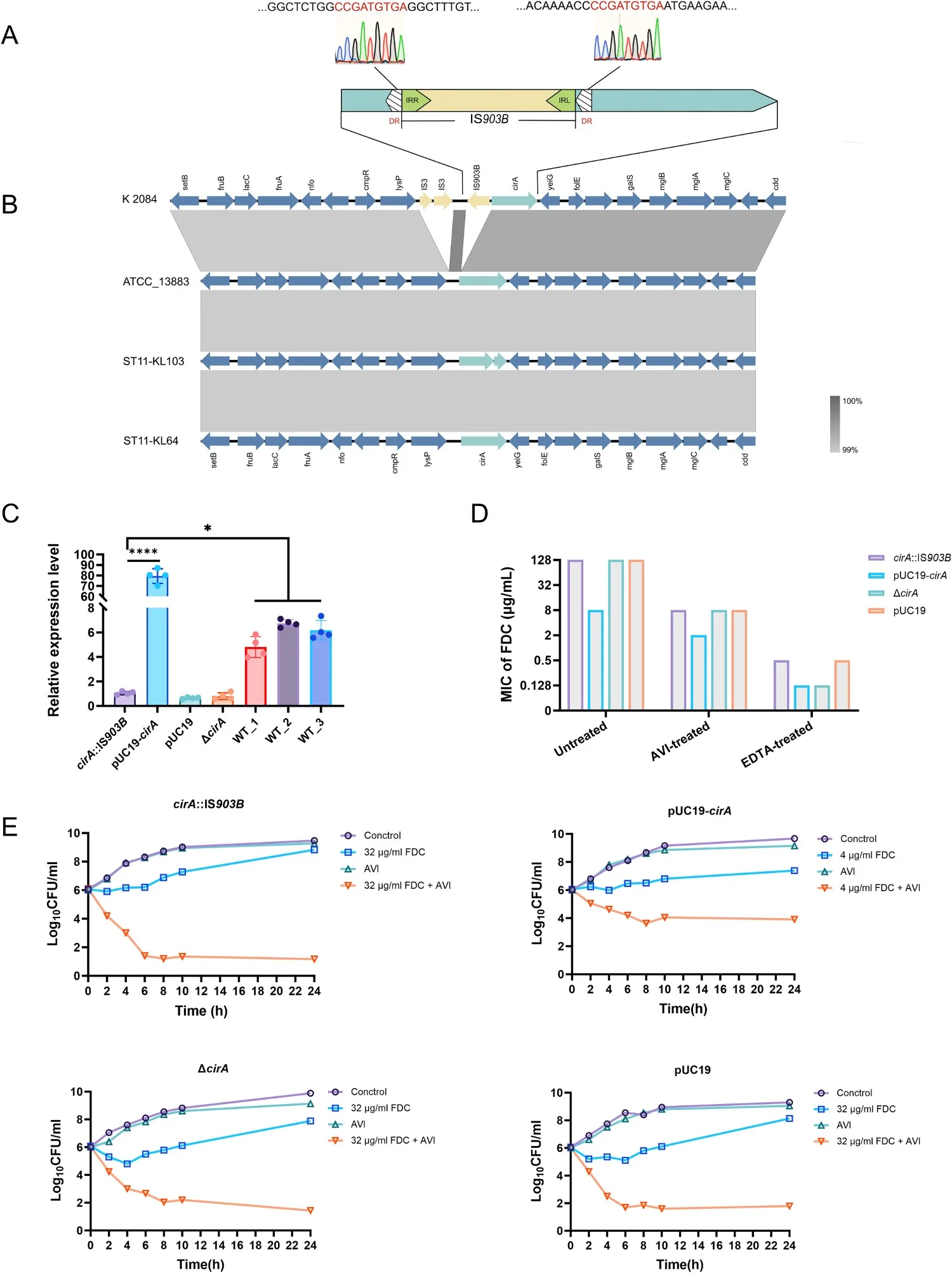
IS*903B* insertion mediates *cirA* inactivation. (A) Sanger sequencing identified the IS*903B* insertion site within the *cirA* gene. The target site duplication (TSD) generated by insertion is highlighted in red. TSD, target site duplication; IRR, right inverted repeat; IRL, left inverted repeat. (B) Synteny analysis of K2084, ATCC 13883, and ST11 strains showing the IS*903B* insertion site and mutation pattern. (C) RT-qPCR analysis of *cirA* expression levels. WT_1, WT_2, and WT_3 represent clinical isolates carrying intact *cirA*. *, *P* < 0.05; ****, *P* < 0.0001. (D) MIC determination and restoration of FDC susceptibility following β-lactamase inhibitor treatment. AVI, avibactam (4 μg/mL); EDTA, ethylenediaminetetraacetic acid (0.4 mM). (E) Time-kill curve analysis under monotherapy and combination treatment conditions. EDTA was not included in time-kill assays because of its limited clinical relevance as a therapeutic agent.

Phylogenetic analysis identified four ST11 strains carrying truncated *cirA* mutations, all belonging to the KL64 or KL103 capsular types. Synteny analysis of the *cirA* locus between K2084 and ST11 reference strains with different KL types (GCF_000240185.1 and GCF_038431215.1) showed that this region was highly conserved across the ST11 lineage, with major differences involving IS insertion patterns. No large-scale genomic rearrangements were observed in this region among different strains, and *cirA* inactivation in K2084 primarily resulted from a local insertion event (Figure 2B).

### *cirA* inactivation cooperates with carbapenemases to drive high-level FDC resistance

To determine whether the contribution of *cirA* inactivation to FDC resistance was broadly conserved, we first observed at the clinical isolate level that strains carrying truncated *cirA* exhibited significantly higher FDC MICs than those of non-truncated isolates. Using the representative strain K2084 (*cirA*::IS*903B*) and its isogenic derivatives as models, RT-qPCR analysis showed that *cirA* expression was markedly reduced in both *cirA*::IS*903B* and the *cirA* deletion mutant (Δ*cirA*) compared with that in clinical wild-type isolates, whereas expression was significantly restored in the complemented strain pUC19-*cirA* (Figure 2C). A low-copy-number complementation system showed a similar trend. Antimicrobial susceptibility testing further demonstrated that both *cirA*::IS*903B* and Δ*cirA* exhibited high-level FDC resistance, whereas the MIC of pUC19-*cirA* decreased markedly to 8 μg/mL.

Combination susceptibility testing further revealed the contribution of β-lactamases to the resistance phenotype. Following the addition of avibactam, the MICs of both *cirA*::IS*903B* and Δ*cirA* decreased 16-fold to 8 μg/mL, whereas the MIC of pUC19-*cirA* decreased only 4-fold to 2 μg/mL. Inhibition of *bla*_NDM_-associated activity by the metal chelator EDTA further reduced the MICs of all strains, with the MICs of *cirA*::IS*903B*, Δ*cirA*, and the complemented strain decreasing to 0.5, 0.128, and 0.128 μg/mL, respectively (Figure 2D). These results indicate that β-lactamase activity further amplifies the level of FDC resistance in the background of *cirA* loss of function.

Time-kill assays showed that FDC alone exerted only limited inhibitory activity, whereas combination treatment with avibactam markedly reduced the bacterial burden, with sustained decreases over 24 h (≥2 log₁₀ CFU/mL) (Figure 2E), indicating significant bactericidal activity. These findings were consistent with the MIC results and further supported a cooperative effect between *cirA* loss of function and β-lactamase activity in mediating FDC resistance, while inhibition of β-lactamase activity substantially restored the bactericidal efficacy of FDC.

In contrast, the susceptibility profiles of *cirA*::IS*903B*, pUC19- *cirA*, and Δ*cirA* strains against other clinically relevant antimicrobial agents, including novel β-lactam/β-lactamase inhibitor combinations and other last-line agents, were comparable (Supplementary Table 3).

### IS*903B*-mediated *cirA* inactivation enhances adaptation under iron-limited conditions and plasmid stability

Given the recurrent independent occurrence of *cirA* loss-of-function events in ST11 high-risk clones, we hypothesized that this event may confer a fitness advantage. To test this hypothesis, the representative strain K2084 (*cirA*::IS*903B*) and its isogenic derivatives were used to compare the physiological performance of *cirA*::IS*903B*, pUC19-*cirA*, and Δ*cirA* under different culture conditions.

In nutrient-rich LB medium, *cirA*::IS*903B* and pUC19-*cirA* showed no significant difference in growth, whereas Δ*cirA* grew more slowly during the first 12 h before subsequently exhibiting a higher growth rate. Under iron-limited conditions, *cirA*::IS*903B* exhibited faster growth, pUC19-*cirA* grew relatively slowly, and Δ*cirA* consistently showed the lowest growth rates (Figure 3A,B). Notably, although both *cirA*::IS*903B* and Δ*cirA* resulted in loss of *cirA* function, they exhibited markedly different growth phenotypes in ID-CAMHB.

**Figure 3.**
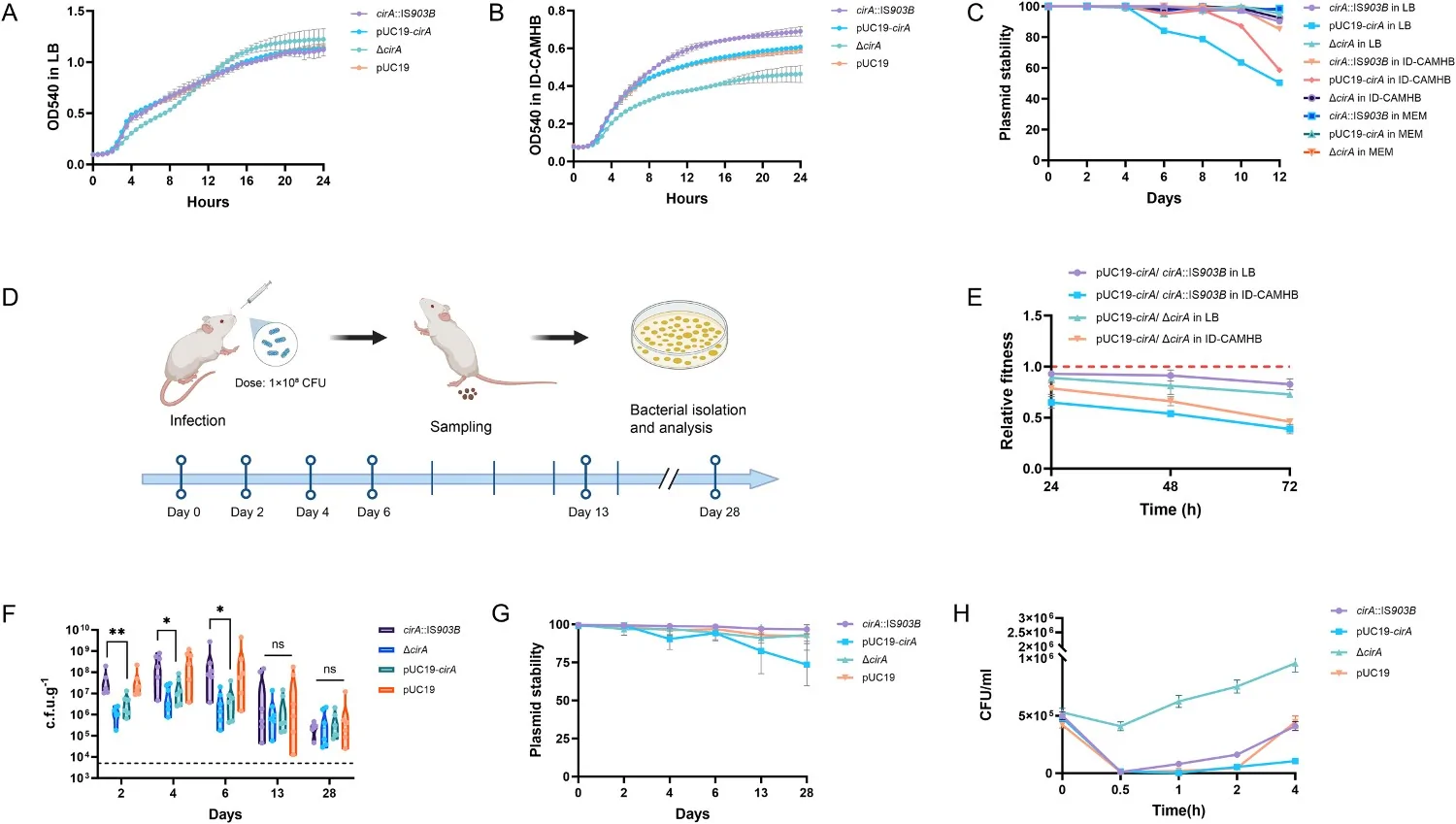
Effects of *cirA* inactivation on bacterial fitness. (A–B) Growth curves of each strain in LB and ID-CAMHB. (C) Retention rates of resistance plasmids during 12-day serial passage in LB, ID-CAMHB, and under antibiotic selection. (D) Bacterial colonization load in the mouse long-term intestinal colonization model, with faecal samples collected on days 2, 4, 6, 13, and 28. (E) Time-course changes in relative fitness (RF) of strains under different culture conditions in in vitro competition assays. (F) Colony counts of strains recovered from mouse faeces. The dashed line indicates the detection limit (5 × 10³ c.f.u./g). *, *P* < 0.05; **, *P* < 0.01. (G) Plasmid stability of resistant isolates recovered from mouse faeces. (H) Intracellular survival of different strains in RAW 264.7 macrophages.

Plasmid stability assays showed that after 14 days of serial passage in the absence of antibiotic pressure, the retention rate of resistance plasmids in *cirA*::IS*903B* was significantly higher than that in pUC19-*cirA* (92.5% vs. 43% in LB, 80.3% vs. 49.3% in ID-CAMHB) and was comparable to that in Δ*cirA* (Figure 3C). Under antibiotic selection, differences among strains were abolished, indicating that *cirA* inactivation may promote the long-term maintenance of resistance plasmids under non-selective conditions. In addition, competition experiments (Figure 3E) showed that the relative fitness (RF) of pUC19-*cirA* compared with *cirA*::IS*903B* and Δ*cirA* gradually declined over time in both LB and ID-CAMHB, with RF values consistently below 1, indicating a fitness cost. This trend was more pronounced under iron-limited conditions, suggesting that *cirA* expression may impose a physiological burden in iron-restricted environments. No significant differences in virulence were observed among the strains (Supplementary Figure 2), and the conjugation efficiency of resistance plasmids remained unchanged.

### IS*903B*-mediated *cirA* inactivation enhances early intestinal colonization and intracellular survival

To evaluate the effect of *cirA* status on *in vivo* colonization, a long-term mouse intestinal colonization model was established, and faecal samples were collected on days 2, 4, 6, 13, and 28 post-inoculation to measure *K. pneumoniae* colonization loads (Figure 3D). As shown in Figure 3F, colonization levels during the early stage of infection ranged from 10⁵ to 10⁸ c.f.u./g. *cirA*::IS*903B* exhibited significantly higher colonization loads than those of pUC19-*cirA* and Δ*cirA* on days 2, 4, and 6. However, by days 13 and 28, colonization levels had declined markedly in all groups, and differences among strains were no longer significant, indicating that the colonization advantage conferred by *cirA*::IS*903B* was primarily observed during the early stage of infection. This early colonization advantage may facilitate the initial establishment of infection and enhance transmission potential within the host.

In addition, analysis of plasmid stability in isolates recovered from mouse faeces (Figure 3G) showed that, under non-selective conditions, plasmid retention rates in *cirA*::IS*903B* and Δ*cirA* were higher than those in the complemented strain pUC19-*cirA*, consistent with the *in vitro* plasmid stability results. These findings further support the conclusion that *cirA* inactivation facilitates the maintenance of resistance plasmids.

To further investigate the role of *cirA* in pathogen–host interactions, intracellular survival assays were performed using RAW 264.7 macrophages (Figure 3H). Intracellular bacterial burdens of all strains decreased markedly during the early stage of infection (0–1 h), followed by varying degrees of recovery. Δ*cirA* exhibited the strongest intracellular survival capacity, with bacterial counts continuously increasing after 1 h and reaching approximately 1.8 times the initial level at 4 h. In contrast, the complemented strain pUC19-*cirA* showed the weakest intracellular survival, recovering to only 22% of the initial level at 4 h. *cirA*::IS*903B* and the empty-vector control pUC19 showed gradual recovery after the initial decline, with intracellular burdens returning to near-baseline levels by 4 h. Notably, *cirA*::IS*903B* and Δ*cirA* displayed distinct intracellular survival patterns, suggesting that different forms of *cirA* inactivation exert differential effects on intracellular adaptation within host cells.

### RNA-seq reveals distinct transcriptional effects associated with different forms of *cirA* inactivation

Transcriptomic sequencing was performed for *cirA*::IS*903B*, pUC19-*cirA*, and Δ*cirA* under both LB and ID-CAMHB conditions.

Transcriptomic comparison of pUC19-*cirA* and *cirA*::IS*903B* in ID-CAMHB identified 73 upregulated and 71 downregulated genes. Differentially expressed genes were mainly enriched in oxidation–reduction processes and transmembrane transport-related functional categories. Among these genes, iron acquisition-associated genes, including *ybtE*, *ybtU*, *ybtT*, and *iutA*, were significantly downregulated. In addition, multiple genes involved in central metabolism and transport showed downregulation trends, including *lldD*, which encodes an FMN-dependent lactate dehydrogenase, NADH redox-associated genes, and the autoinducer-2 (AI-2) transport system genes *lsrABCD*, together with its regulatory factor *lsrR*.

KEGG pathway enrichment analysis further identified sulphur metabolism (rich factor = 0.44, adjusted *P* = 0.0022) and arginine/proline metabolism (rich factor = 0.22, adjusted *P* = 0.0003) as significantly enriched pathways. In the complemented strain, genes involved in sulphate uptake and assimilation, including *cysA/C/D/G/N/K/P*, were significantly downregulated. These genes are associated with Fe-S cluster biosynthesis, and the *suf* gene cluster involved in Fe-S cluster assembly and delivery also showed a downregulation trend. RT-qPCR analysis of *sufS* and *sufD* further supported this expression pattern (Figure 4E). In addition, genes associated with arginine/proline metabolism, including *astA/B/C/D/E*, also exhibited downregulation trends (Figure 4A).

**Figure 4.**
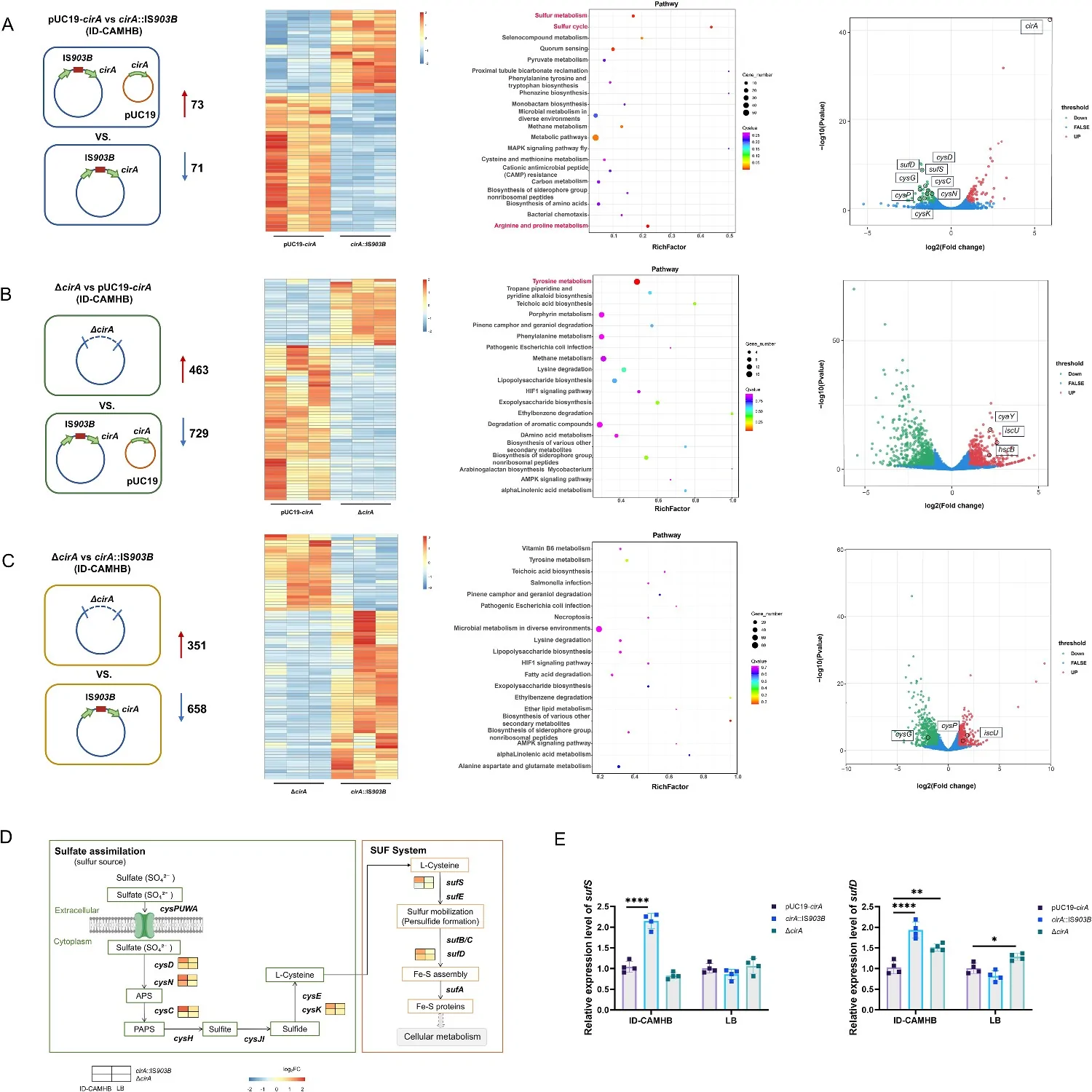
Transcriptomic differences associated with IS*903B* insertion and *cirA* deletion under iron-limited conditions. (A–C) Heatmaps, KEGG enrichment analyses, and volcano plots of differentially expressed genes under iron-limited conditions for comparisons between pUC19-*cirA* and *cirA*::IS*903B*, Δ*cirA* and pUC19-*cirA*, and Δ*cirA* and *cirA*::IS*903B*, respectively. (D) Expression changes of genes involved in assimilatory sulphate reduction and Fe-S cluster biosynthesis in *cirA*::IS*903B* and Δ*cirA* relative to pUC19-*cirA* under ID-CAMHB and LB conditions. (E) Relative expression levels of *sufS* and *sufD* in pUC19-*cirA*, *cirA*::IS*903B*, and Δ*cirA*. *, *P* < 0.05; **, *P* < 0.01; ****, *P* < 0.0001. Functional clustering of differentially expressed genes suggested potential involvement of Fe-S-associated metabolic pathways, although the underlying mechanisms require further investigation.

In ID-CAMHB, comparison of Δ*cirA* with pUC19-*cirA* identified 463 upregulated and 729 downregulated genes. Fe-S-associated genes were significantly upregulated in Δ*cirA*, including the Fe-S cluster scaffold protein gene *iscU*, the chaperone protein gene *hscB*, the accessory factor gene *cyaY*, and the gene encoding the iron starvation-associated electron carrier flavodoxin (Figure 4B). In parallel, multiple siderophore-mediated iron acquisition systems, including *fyuA*, *ybtP*, *iucC*, *iutA*, *entC*, and *fepA*, were upregulated, suggesting enhanced iron uptake under iron-restricted conditions. KEGG enrichment analysis identified tyrosine metabolism as the most significantly enriched pathway (rich factor = 0.49, adjusted *P* = 0.039). In addition, several genes associated with energy metabolism, ATP-binding cassette transport, and quorum sensing, including *lsrA*, *bcsB*, and *ecpA*, showed upregulation trends in Δ*cirA*. Expression of plasmid maintenance-associated genes, including *hipA*, *vapC*, and *mukB*, was also increased.

To further compare the transcriptional responses associated with different forms of *cirA* inactivation, *cirA*::IS*903B* and Δ*cirA* were directly compared (Figure 4C). In ID-CAMHB, Fe-S cluster assembly-associated genes, including *iscU*, showed higher expression levels in Δ*cirA* than in the IS insertion strain, whereas several sulphur metabolism-associated genes, including members of the *cys* family, exhibited distinct expression patterns between the two mutant backgrounds, suggesting differential regulation of Fe-S-associated and related metabolic pathways. Notably, further analysis of genes within the 20-kb region upstream and downstream of the *cirA* locus revealed no consistent local transcriptional alterations in *cirA*::IS*903B* compared with the complemented strain pUC19-*cirA*. These findings suggest that the observed global transcriptional differences are unlikely to result from simple local cis-regulatory effects on neighbouring genes.

Taken together, *cirA*::IS*903B* and Δ*cirA* exhibited distinct transcriptional profiles related to Fe-S-associated and iron acquisition systems in ID-CAMHB, suggesting that different forms of *cirA* inactivation may be associated with distinct metabolic states and adaptive outputs. These transcriptional differences were not evident under LB conditions or in the empty-vector control background (Supplementary Figure 3).

Consistent with the transcriptomic analysis identifying *sufS* as a candidate mediator of the adaptive response to iron limitation, deletion of *sufS* in the K2084 (*cirA*::IS*903B*) background significantly impaired the iron-limitation-associated adaptive phenotypes. Compared with the parental *cirA*::IS*903B* strain, the Δ*sufS* mutant exhibited reduced growth in iron-depleted medium, reduced resistance plasmid stability, and diminished competitive fitness under iron-limited conditions. Complementation of *sufS* restored these phenotypes to levels comparable to those of the parental strain (Figure 5A–C). These results indicate that *sufS* plays an important role in maintaining the adaptive advantages conferred by IS*903B*-mediated *cirA* inactivation under iron-limited conditions.

**Figure 5.**
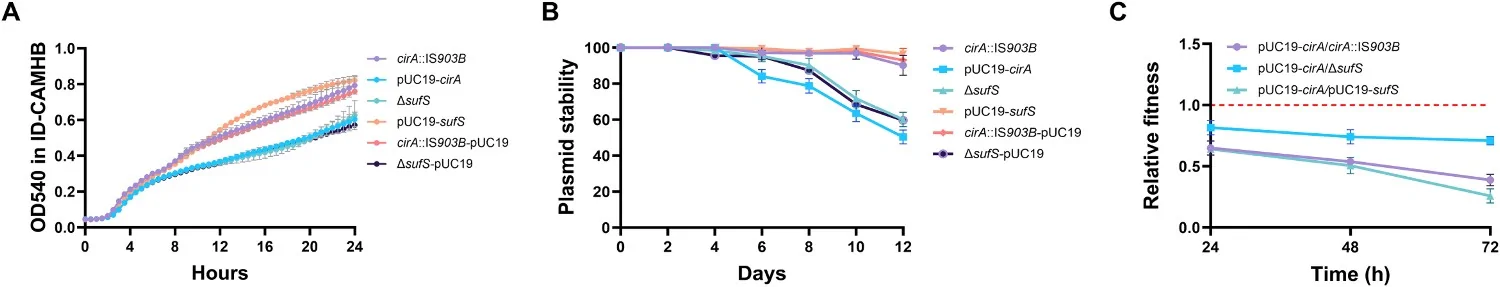
Genetic validation of SufS-mediated contribution to iron-limitation-associated adaptation in the *cirA*::IS*903B* background. (A) Growth curves of the *cirA*::IS*903B* strain, the Δ*sufS* mutant, and the corresponding *sufS*-complemented strain in iron-depleted ID-CAMHB medium. (B) Resistance plasmid stability of the indicated strains. (C) Competitive fitness of the indicated strains under iron-limited conditions. The Δ*sufS* mutant and its corresponding complemented strain (pUC19*-sufS*) were constructed in the K2084 background, while empty-vector controls (*cirA*::IS*903B*-pUC19 and Δ*sufS*-pUC19) were included as indicated.

### IS*903B*-mediated *cirA* inactivation is associated with enhanced antioxidant capacity

Transcriptomic analysis showed that, in ID-CAMHB, Fe-S-associated metabolic pathways and related genes were generally downregulated in the complemented strain relative to *cirA*::IS*903B*. Previous studies have shown that iron limitation can affect Fe-S-associated metabolism and can be accompanied by alterations in cellular oxidative stress status [28,29], whereas the assimilatory sulphate reduction pathway participates in redox balance and stress adaptation. In parallel, several sulphur metabolism-associated genes, including *cysND*, *cysC*, and *cysI*, exhibited marked expression changes (Figure 4D), suggesting distinct oxidative stress response features among strains with different *cirA* states. Based on these findings, aconitase activity under iron-limited conditions was further evaluated among different strains. As shown in Figure 6A, *cirA*::IS*903B* exhibited the highest aconitase activity (0.24 ± 0.01), whereas aconitase activity was significantly reduced in the complemented strain (0.11 ± 0.01). In contrast, both Δ*cirA* and the empty-vector control pUC19 maintained relatively high aconitase activity.

**Figure 6.**
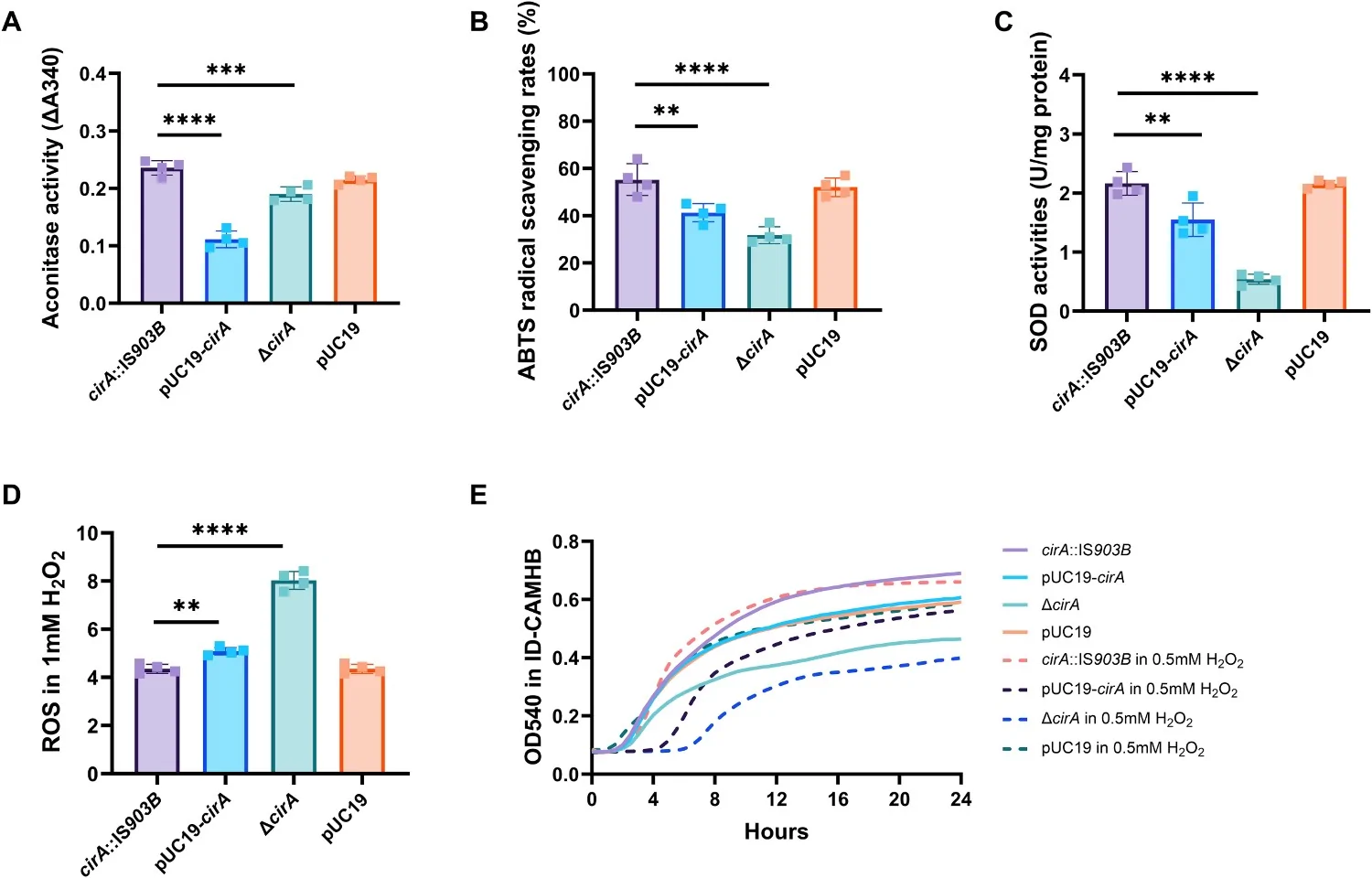
Metabolic and oxidative stress-associated phenotypes related to *cirA* inactivation. (A) Aconitase activity of different strains under iron-limited conditions. (B) Comparison of ABTS radical scavenging activity. (C) SOD activity assay. (D) ROS levels of different strains under 1 mM H₂O₂ treatment. (E) Growth curves of different strains in ID-CAMHB supplemented with 0.5 mM H₂O₂. **, *P* < 0.01; ***, *P* < 0.001; ****, *P* < 0.0001.

Oxidative stress-associated phenotypes were subsequently evaluated among strains carrying different *cirA* states. 2,2′-azinobis-(3-ethylbenzothiazoline-6-sulfonic acid) (ABTS) radical scavenging assays showed that restoration of *cirA* reduced the overall antioxidant capacity relative to the wild-type strain (*P* < 0.05), whereas a more pronounced reduction was observed in Δ*cirA* (*P* < 0.001) (Figure 6B). Consistently, superoxide dismutase (SOD) activity decreased in the complemented strain (1.55 ± 0.28 vs 2.16 ± 0.20) and was further reduced in Δ*cirA* (0.54 ± 0.08 vs 2.16 ± 0.20) (Figure 6C). Under 1 mM H₂O₂ treatment, intracellular reactive oxygen species (ROS) levels were significantly increased in both pUC19-*cirA* (5.09 ± 0.15 vs 4.35 ± 0.17) and Δ*cirA* (8.03 ± 0.39 vs 4.35 ± 0.17) (Figure 6D). Similarly, under 0.5 mM H₂O₂ treatment, the growth rates of pUC19-*cirA* and Δ*cirA* were markedly lower than those of the control strain (Figure 6E). These findings indicate that strains carrying different *cirA* states exhibit distinct antioxidant capacities and oxidative stress response phenotypes.

To further validate the phenotypes associated with *cirA*::IS*903B*, *cirA*::IS*903B* derivatives were constructed by replacing the endogenous *cirA* in ST11 clinical isolates carrying *bla*_KPC_ or *bla*_NDM_, followed by growth competition assays under iron-limited conditions. The constructed strains similarly exhibited growth competition advantages in ID-CAMHB (Supplementary Figure 4), generally consistent with the iron-limited adaptation trend observed in the K2084 background.

## Discussion

As an important last-resort agent against CRKP [30], resistance to FDC is of particular concern. However, whether resistance-associated mutations simultaneously affect bacterial adaptation within host environments remains unclear. By integrating global genomic analysis with functional experiments, this study demonstrated that IS*903B*-mediated *cirA* inactivation not only confers high-level FDC resistance but is also associated with enhanced growth competition under iron-limited conditions, increased resistance plasmid stability, and improved host-associated survival, without marked changes in virulence or plasmid conjugation ability. These findings suggest that certain last-resort resistance mechanisms may persist without imposing substantial fitness costs, thereby increasing the risk of long-term maintenance and dissemination of high-risk CRKP clones.

CirA is an iron transport-associated receptor, and its loss of function has been shown to reduce FDC susceptibility and to be associated with NDM production [14,17,31]. Based on analysis of NCBI genomes, this study found that *cirA* loss-of-function mutations were more frequently detected in strains carrying *bla*_KPC_ or *bla*_NDM_, suggesting that highly resistant genetic backgrounds may impose synergistic selective pressure on their evolution. Although global *cirA* variation was predominantly characterized by amino acid substitutions and loss-of-function mutations remained relatively rare overall, these mutations showed a temporal accumulation trend among isolates from China and emerged independently multiple times across distinct ST11 phylogenetic branches. This convergent evolutionary pattern suggests that, in highly resistant genetic backgrounds carrying *bla*_KPC_/*bla*_NDM_, *cirA* inactivation may be more readily generated and retained. Previous studies have shown that inhibition of NDM activity reduces the emergence of *cirA* mutations [32], further supporting the selective role of resistance gene backgrounds in their evolution. Collectively, *cirA* inactivation may represent an adaptive evolutionary pathway repeatedly selected under specific selective pressures, such as iron-limited environments, rather than being driven by the expansion of a single clone.

Unlike previous studies that primarily focused on the epidemiological distribution and mutational characteristics of defective CirA [33], this study further compared the fitness phenotypes and transcriptional responses associated with different forms of *cirA* inactivation, suggesting that distinct mechanisms of *cirA* loss of function may result in different biological consequences. In functional experiments, both K2084 (*cirA*::IS*903B*) and Δ*cirA*, in which the entire IS*903B*-*cirA* region was deleted, exhibited reduced *cirA* transcription and FDC resistance, but displayed markedly different adaptive phenotypes. Notably, neither strain exhibited detectable collateral sensitivity to other clinically relevant antimicrobial agents. Δ*cirA* showed impaired growth under iron-limited conditions, consistent with previous reports [15,34]. In contrast, *cirA*::IS*903B* exhibited enhanced growth and adaptive capacity. As Δ*cirA* lacked both IS*903B* and *cirA*, whereas K2084 retained IS*903B* despite *cirA* inactivation, these findings suggest that the presence of IS*903B* within the *cirA*-inactivated background may be associated with adaptive features distinct from those of Δ*cirA*. In addition, *cirA*::IS*903B* derivatives constructed in multiple independent ST11 clinical isolates similarly exhibited growth competition advantages under iron-limited conditions, further suggesting that this phenotype may not be restricted to a single strain background.

Iron-sulphur (Fe-S) clusters play critical roles in redox reactions and gene regulation [35,36]. In this study, aconitase, an Fe-S-dependent enzyme, exhibited the highest activity in the IS*903B*-mediated *cirA*-inactivated strain under iron-limited conditions, whereas its activity was significantly reduced following *cirA* complementation [37]. These findings functionally support the presence of distinct Fe-S-associated metabolic features among strains with different *cirA* states. In addition, deletion of *sufS* in the *cirA*::IS*903B* background attenuated the associated adaptive phenotypes, further suggesting a potential contribution of Fe-S-associated metabolism to this adaptive state. Previous studies have linked the Suf system to bacterial intestinal colonization and intracellular survival [38,39]. Consistent with these observations, *cirA*::IS*903B* exhibited enhanced early intestinal colonization and macrophage intracellular survival. In addition, Fe-S-associated proteins are closely linked to cellular redox homeostasis [40–42]. Downregulation of Fe-S-associated genes in the complemented strain was accompanied by increased ROS levels, suggesting that different forms of *cirA* inactivation may be associated with distinct oxidative stress adaptation phenotypes. Notably, the Fe-S-associated transcriptional profile of Δ*cirA* under iron-limited conditions was not fully consistent with that of *cirA*::IS*903B*, further indicating that different forms of *cirA* inactivation may involve distinct metabolic adaptation states. Overall, the adaptive advantages and enhanced plasmid stability observed in *cirA*::IS*903B* may facilitate the persistence of related strains in both host and clinical environments.

This study still has several limitations. Functional analyses were primarily based on isogenic strain construction and *cirA*::IS*903B* derivatives generated in multiple ST11 backgrounds, which facilitated comparison of phenotypic differences associated with distinct forms of *cirA* inactivation, but did not encompass other sequence types or *cirA* variants mediated by different IS elements. In addition, the specific regulatory mechanisms linking Fe-S-associated metabolic alterations with the adaptive phenotypes observed in *cirA*::IS*903B* remain incompletely understood and require further validation through integrated multi-omics approaches.

Taken together, although both *cirA*::IS*903B* and Δ*cirA* resulted in *cirA* loss of function, the two strains exhibited distinct adaptive phenotypes under iron-limited conditions, suggesting that certain last-resort resistance mechanisms may not impose substantial fitness costs. Notably, the representative strain investigated in this study already exhibited high-level FDC resistance despite the absence of prior FDC exposure in the patient, indicating that such resistance-associated mutations may emerge and be retained without direct antibiotic selection. With the increasing clinical use of FDC, continued surveillance of these high-risk CRKP clones is warranted. The adaptive advantages and enhanced plasmid stability associated with *cirA*::IS*903B* may further facilitate the persistence of resistant strains in both host and clinical environments, thereby increasing the difficulty of eradication and antimicrobial treatment. Incorporating assessment of *cirA* integrity and IS insertion status into CRKP genomic surveillance may aid in the early identification of FDC-resistant clones with dissemination potential.

## Supplementary Material

S2.tif

S3.tif

S4.tif

Graphical Abstract.tif

S1.tif

Supplementary.docx

## Data Availability

Complete sequences of the *K. pneumoniae* isolate has been deposited on NCBI with BioProject no. SAMN52642892.
