# Supplementary material for: IS903B-mediated cirA inactivation confers cefiderocol resistance and adaptive fitness in carbapenem-resistant Klebsiella pneumoniae
Source: Emerg Microbes Infect. 2026 Sep 25;15(1):2736341. doi: 10.1080/22221751.2026.2736341 (PMC13618097; doi:10.1080/22221751.2026.2736341)
Supplement: Supplementary file 1 [file TEMI_A_2736341_SM5749.docx]

**Supplementary Methods**

**Construction of *cirA* deletion and complementation strains**

Targeted gene deletion was performed using the CRISPR/Cas9 genome editing system. Briefly, the pCasKP plasmid was introduced into the target strain K2084 and the ST11 clinical isolates Kp1 and Kp2 by electroporation to enable Cas9 expression. Spacer sequences were designed using the Cas-Designer tool ([http://www.rgenome.net/cas-designer/](http://www.rgenome.net/cas-designer/)), and the corresponding target fragments were amplified by PCR. The pSGKP plasmid was then digested with restriction enzymes, and the amplified spacer fragments were ligated into the linearized vector to generate recombinant plasmids carrying the spacer sequences. The ligation products were transformed into chemically competent *Escherichia coli*, and positive clones were selected on hygromycin-containing plates (100 μg/mL) and confirmed by sequencing.

The verified recombinant plasmids were subsequently mixed with donor DNA fragments and electroporated into the Cas9-expressing target strain. Gene deletion mutants were screened on plates containing hygromycin and apramycin (50 μg/mL), followed by sequencing verification. During the screening process, antibiotic selection combined with PCR confirmation was performed to ensure successful deletion of the target region. Plasmid curing was finally carried out on sucrose-containing plates.

For complementation, the wild-type *cirA* gene was cloned into the pUC19 plasmid and introduced into the *cirA*-deficient strains by electroporation. Transformants were cultured on LB agar plates supplemented with chloramphenicol (50 μg/mL), and the presence of *cirA* was confirmed by PCR. To preliminarily evaluate whether the phenotypes associated with *cirA*::IS*903B* were reproducible in additional strain backgrounds, the *cirA* allele carrying the IS*903B* insertion was further introduced into an ST11 clinical isolate harboring intact *cirA*. Growth and competition capacity under iron-limited conditions were subsequently assessed. Primer sequences are listed in Supplementary Table 1.

**Whole-genome sequencing**

Genomic DNA was extracted using the TIANamp Bacterial DNA Kit (TIANGEN Biotech, Beijing, China). Whole-genome sequencing was performed using the Illumina HiSeq X Ten platform (Illumina, USA) and the PacBio Sequel II system (Pacific Biosciences, USA). Raw sequencing reads were quality-filtered using Trimmomatic v0.3, and short-read assemblies were generated using SPAdes v3.15.4 [1]. Hybrid assembly was performed using Unicycler v0.5.0 [2]. Phylogenetic trees were constructed using the maximum-likelihood method and visualized and annotated using tvBOT [3]. Comparative genomic maps were generated using BRIG or Easyfig. ISs and other mobile genetic elements were predicted using VRprofile [4].

**Transcriptomic analysis**

Total RNA was extracted from bacterial samples, and ribosomal RNA was removed to reduce background interference. The purified RNA was fragmented into approximately 200-nt fragments using fragmentation buffer. First-strand cDNA synthesis was performed using random hexamer primers, buffer, dNTPs, RNase H, and DNA polymerase I. During second-strand cDNA synthesis, dUTP was incorporated in place of dTTP. The resulting cDNA products were subjected to end repair, A-tailing, and adaptor ligation. To preserve strand-specific information, the second cDNA strand synthesized with dUTP was selectively degraded using uracil-N-glycosylase (UNG). The libraries were subsequently amplified by PCR to enrich nucleic acid fragments and complete library construction. Finally, the prepared libraries were sequenced on the Illumina platform to generate transcriptomic data.

***Galleria mellonella* infection assay**

Healthy *Galleria mellonella* larvae weighing 250-300 mg were randomly assigned to groups of 10 larvae each. Overnight bacterial cultures were diluted in PBS to a final concentration of 1 × 10⁶ CFU/mL. A 10 μL bacterial suspension was injected into the left posterior proleg of each larva using a microsyringe. Control larvae received an equal volume of PBS. Multiple parallel groups were included for each strain.

Following infection, larvae were incubated at 37°C in the dark, and survival was monitored every 12 h for 48 h. Survival data were recorded to generate Kaplan-Meier survival curves. *Klebsiella pneumoniae* strains NTUH-K2044 and ATCC 700603 were used as hypervirulent and low-virulence control strains, respectively.

**Biofilm formation assay**

Biofilm formation was evaluated using a crystal violet staining assay. Bacterial cultures adjusted to the desired concentration were inoculated into 96-well polystyrene microplates at 200 μL per well. Wells containing LB broth alone were included as blank controls. Three biological replicates were performed for each strain.

Microplates were incubated statically at 37°C for 48 h. After incubation, wells were gently washed with PBS to remove planktonic cells and subsequently stained with crystal violet. The bound dye was then dissolved with absolute ethanol for 10–15 min, and absorbance was measured at 540 nm to quantify biofilm biomass.

**Serum resistance assay**

Fresh single colonies of the tested strains were inoculated into 5 mL LB broth and cultured at 37°C with shaking at 150 rpm until the exponential growth phase. Bacterial suspensions were adjusted to approximately 1 × 10⁶ CFU/mL. Subsequently, 25 μL of bacterial suspension was mixed with 75 μL of pooled healthy human serum and incubated at 37°C with shaking.

Samples were collected at 0, 60, 120, and 180 min. At each time point, 10 μL of the reaction mixture was serially diluted 10-fold in LB broth and plated onto LB agar plates. Plates were incubated at 37°C for 16-18 h, followed by colony-forming unit (CFU) enumeration to evaluate serum survival capacity. All experiments were performed with three independent biological replicates.

**Conjugation assay**

Conjugation experiments were performed to evaluate the transferability of resistance plasmids. Donor strains and the recipient strain *Escherichia coli* J53 were cultured overnight in LB broth at 37°C with shaking at 180 rpm. Subsequently, 300 μL of donor culture and 600 μL of recipient culture were mixed in 5 mL fresh LB broth.

The mixed cultures were centrifuged at 3,800 rpm for 2 min, and the supernatant was discarded. The bacterial pellet was resuspended in a small volume of medium, and 20 μL of the suspension was plated onto LB agar plates containing the appropriate dual antibiotics for selection of transconjugants. Plates were incubated at 37°C for 18-24 h, and conjugation frequencies were calculated based on the number of transconjugant colonies obtained.

**Intestinal colonization model**

Bacterial cultures were grown to the exponential phase, washed with PBS, and resuspended to approximately OD600 = 1.0. Each mouse was orally gavaged with 100 μL of bacterial suspension containing 1 × 10⁸ c.f.u. Fecal samples were collected on days 2, 4, 6, 13, and 28 post-inoculation, resuspended in PBS, serially diluted, and plated onto Simmons citrate agar supplemented with 1% inositol (SCAI) for colony enumeration. Bacterial burdens were calculated as c.f.u./g of feces. Six mice were included in each group [5]. In addition, plasmid stability of the recovered isolates was assessed on day 6 post-inoculation. All animal experiments were approved by the Ethics Committee of the First Affiliated Hospital of Nanchang University.

**Macrophage phagocytosis assay**

Mouse macrophage RAW 264.7 cells were used for the assay [6]. Cells were cultured in DMEM (Thermo Fisher Scientific) supplemented with 10% heat-inactivated fetal bovine serum at 37°C with 5% CO₂. Before infection, cells were seeded into 24-well plates at 5 × 10⁵ cells per well (1 mL/well) and cultured for 16 h until reaching 70-80% confluence. Bacteria were then added at a multiplicity of infection (MOI) of 10:1 and co-incubated for 0.5, 1, 2, 4, and 6 h. After incubation, cells were washed with PBS and incubated for 30 min in fresh medium containing 50 μM polymyxin B. Following PBS washing, cells were lysed with 0.1% saponin. Lysates were serially diluted and plated onto LB agar plates for colony counting to assess intracellular bacterial survival.

**Oxidative stress-associated phenotypes and aconitase activity assays**

2,2′-azinobis-(3-ethylbenzothiazoline-6-sulfonic acid) (ABTS) radical scavenging assays and superoxide dismutase (SOD) activity assays were performed to evaluate the antioxidant capacity of the tested strains. Briefly, bacterial cultures collected during the exponential growth phase were subjected to ultrasonic disruption for total protein extraction, and protein concentrations were determined using an enhanced BCA protein assay kit (Beyotime, China). ABTS radical scavenging activity and SOD activity were measured according to the manufacturers’ instructions using commercial assay kits (Solarbio, China). Intracellular reactive oxygen species (ROS) levels and aconitase activity were determined using the corresponding commercial kits (Pumoke, China) following the manufacturers’ protocols.

**Supplementary table 1.** Primers uses in the study

| Name of primers | Sequence （5’-3’） | Name of primers | Sequence （5’-3’） |
| --- | --- | --- | --- |
| 16sRNA-F | ATCGAGGAACGGAACCAACC | *cirA*-KO-R1 | GAAACGCCAACGATACGACGGA |
| 16sRNA-R | GTGGATTCATCGCAGTTGCC | *cirA*-KO-F2 | TCCGTCGTATCGTTGGCGTTTCGGACGATTGGTCCTTCTACGTGA |
| q*cirA*-F | ACTTTGTCGGCTGGAAAACCG | *cirA*-KO-R2 | CAGAAGCGATAATCCACCGCCATA |
| q*cirA*-R | GTCACGTAGAAGGACCAATCGTCC | *sufS*-spacer-F | TAGTTACGGCCCGGCGCAGCGTAA |
| *cirA*-F | CCACATCGACGAGAAAACAGCG | *sufS*-spacer-R | AAACTTACGCTGCGCCGGGCCGTA |
| *cirA*-R | CAGAAGCGATAATCCACCGCCATA | *sufS*-KO-F1 | TTCAGTAGAACAGGTTCGCGCC |
| q*sufD*-F | TCGATGGCCGCTATCATCCT | *sufS*-KO-R1 | CGGACGAACACCACTTCCT |
| q*sufD*-R | TGGGCGGTATTCAGCGCAT | *sufS*-KO-F2 | AGGAAGTGGTGTTCGTCCGGCAGCGTAAAGGGGTAATTGCT |
| q*sufS*-F | CGCCGGGGATAACATCATCATC | *sufS*-KO-R2 | ACCCTAACAGTTTGCGGATCC |
| q*sufS*-R | TGCTGATGCGCAAGAGCAATCA | pCasKP-conf-F | GGGAAATACAGACCGCCACA |
| *cirA*-spacer-F | TAGTCATCAGCGTCATCACCCAAC | pCasKP-conf-R | TGAAGCTGATAGGGGAGCCT |
| *cirA*-spacer-R | AAACGTTGGGTGATGACGCTGATG | pSGKP-conf-F | AGGATTTGCAGACTACGGGC |
| *cirA*-KO-F1 | CCACATCGACGAGAAAACAGCG | pSGKP-conf-R | TGTGTGGAATTGTGAGCGGA |

**Supplementary Table 2.** List of all *K. pneumoniae* isolates with *cirA* loss-of-function mutations identified in the global dataset (n = 21)

| **Assembly accession** | **BioSample** | **MLST** | **KL_type** | ***cirA* status** | **Collection date** | **Geographic location** | **Carbapen-emase** | **No. of plasmids** |
| --- | --- | --- | --- | --- | --- | --- | --- | --- |
| GCF_000240185.1 | SAMN02602959 | 11 | KL103 | interruption | 2011 | China | KPC | 6 |
| GCF_000465975.2 | SAMN02470575 | 29 | KL54 | premature termination | 2015 | Australia | \ | 1 |
| GCF_010586985.1 | SAMN11618737 | 10 | KL10 | premature termination | missing | USA | \ | 3 |
| GCF_020911785.1 | SAMN23002891 | 2643 | KL23 | premature termination | missing | China | \ | 0 |
| GCF_020911805.1 | SAMN23002890 | 23 | KL1 | interruption | missing | China | \ | 0 |
| GCF_963854595.1 | SAMEA114785897 | 6260 | KL17 | interruption | 2023 | Bulgaria | NDM | 5 |
| GCF_023573885.1 | SAMN28095478 | 11 | KL64 | premature termination | 2022 | China | KPC | 5 |
| GCF_030388445.1 | SAMN34122257 | 14 | KL64 | premature termination | 2021 | United Arab Emirates | NDM | 7 |
| GCF_017743115.1 | SAMN18450112 | 660 | KL16 | interruption | 2020 | China | \ | 1 |
| GCF_023373545.1 | SAMN20856063 | 5214 | KL1 | premature termination | 2019 | China | NDM | 4 |
| GCF_015138775.1 | SAMD00196002 | 20 | KL28 | partial deletion | 2018 | Japan | \ | 5 |
| GCF_015910145.1 | SAMN13747524 | 376 | KL2 | interruption | 2014 | Canada | \ | 6 |
| GCF_020149895.1 | SAMEA3357330 | 359 | KL10 | premature termination | 2013 | Australia | \ | 6 |
| GCF_030020765.1 | SAMN34258006 | 380 | KL2 | interruption | 2022 | United Kingdom | \ | 3 |
| GCF_039518015.1 | SAMN41106943 | 11 | KL64 | premature termination | 2021 | China | KPC | 4 |
| GCF_009661415.2 | SAMN13153760 | 26 | KL10 | premature termination | 2018 | Russia | \ | 5 |
| GCF_013704985.1 | SAMN15148656 | 359 | KL28 | premature termination | 2017 | United Kingdom | \ | 6 |
| GCF_014725775.1 | SAMN16085534 | 86 | KL2 | premature termination | 2015 | Singapore | KPC | 3 |
| GCF_038431215.1 | SAMN36766249 | 11 | KL64 | partial deletion | 2014 | China | \ | 4 |
| GCF_001708225.1 | SAMN05425526 | 23 | KL1 | insertion | 2016 | China | \ | 1 |
| GCF_900635995.1 | SAMEA3368254 | 375 | KL2 | premature termination | 1911 | USA | \ | 3 |

**Supplementary table 3.** Antimicrobial susceptibility profiles of the tested *Klebsiella pneumoniae* strains

| Strains | VITEK-2 system | | | | | | | | E-test | | |
| --- | --- | --- | --- | --- | --- | --- | --- | --- | --- | --- | --- |
|  | AMK | MNO | COL | LVX | TGC | IPM | MEM | ATM | M/V | I/R | CZA |
| *cirA*::IS*903B* | ≥64 | ≥16 | ≤0.5 | ≥8 | ≥8 | ≥16 | ≥16 | ≥64 | 16 | 2 | ≥16 |
| pUC19-*cirA* | ≥64 | ≥16 | ≤0.5 | ≥8 | ≥8 | ≥16 | ≥16 | ≥64 | 16 | 2 | ≥16 |
| Δ*cirA* | ≥64 | ≥16 | ≤0.5 | ≥8 | ≥8 | ≥16 | ≥16 | ≥64 | 16 | 2 | ≥16 |
| pUC19 | ≥64 | ≥16 | ≤0.5 | ≥8 | ≥8 | ≥16 | ≥16 | ≥64 | 16 | 2 | ≥16 |

AMK: Amikacin, MNO: Minocycline, COL: Colistin, LVX: Levofloxacin, TGC: Tigecycline, IPM: Imipenem, MEM: Meropenem, ATM: Aztreonam, M/V: Meropenem/Vaborbactam, I/R: Imipenem/Relebactam, CZA: Ceftazidime/avibactam

**Supplementary Figure 1. Core genome phylogeny of ST11 strains.** The core genome phylogenetic tree illustrates the branching structure of 478 ST11 isolates and is annotated with capsular serotype, carbapenemase type, geographic origin, and collection year. Four strains carrying *cirA* loss-of-function mutations are highlighted with red circles and are distributed across distinct phylogenetic branches.

**Supplementary Figure 2. Comparison of virulence-associated phenotypes among different strains.** A. *Galleria mellonella* infection assay.B. Human serum resistance assay. C. Crystal violet staining assay for biofilm formation capacity.

**Supplementary Figure 3. Transcriptomic differential expression analysis under different culture conditions.** A. KEGG enrichment analysis and volcano plot of differentially expressed genes in the complemented strain pUC19-*cirA* relative to the wild-type strain *cirA*::IS*903B* under LB conditions. B. KEGG enrichment analysis and volcano plot of differentially expressed genes in the empty-vector strain pUC19 relative to the wild-type strain *cirA*::IS*903B* under ID-CAMHB conditions.

**Supplementary Figure 4. Validation of adaptive phenotypes associated with *cirA*::IS*903B* derivatives in different ST11 clinical strain backgrounds.** A-B. Growth curves of Kp1- and Kp2-background strains under ID-CAMHB conditions. C. Resistance plasmid stability during serial passage in LB medium. D. Competition assays of Kp1- and Kp2-background strains under ID-CAMHB conditions. The dashed line indicates a relative fitness value of 1. Data are presented as mean ± SD.

[1]	Prjibelski A, Antipov D, Meleshko D, et al. Using SPAdes De Novo Assembler. Curr Protoc Bioinformatics. 2020;70(1):e102.

[2]	Wick RR, Judd LM, Gorrie CL, et al. Unicycler: Resolving bacterial genome assemblies from short and long sequencing reads. PLoS Comput Biol. 2017;13(6):e1005595.

[3]	Xie J, Chen Y, Cai G, et al. Tree Visualization By One Table (tvBOT): a web application for visualizing, modifying and annotating phylogenetic trees. Nucleic Acids Res. 2023;51(W1):W587-w592.

[4]	Li J, Tai C, Deng Z, et al. VRprofile: gene-cluster-detection-based profiling of virulence and antibiotic resistance traits encoded within genome sequences of pathogenic bacteria. Brief Bioinform. 2018;19(4):566-574.

[5]	Wei DW, Song Y, Li Y, et al. Insertion sequences accelerate genomic convergence of multidrug resistance and hypervirulence in Klebsiella pneumoniae via capsular phase variation. Genome Med. 2025;17(1):45.

[6]	Jia X, Zhu Y, Jia P, et al. The key role of iroBCDN-lacking pLVPK-like plasmid in the evolution of the most prevalent hypervirulent carbapenem-resistant ST11-KL64 Klebsiella pneumoniae in China. Drug Resist Updat. 2024;77:101137.
